# Adding Value to Liquid Biopsy for Brain Tumors: The Role of Imaging

**DOI:** 10.3390/cancers15215198

**Published:** 2023-10-29

**Authors:** Nastaran Khalili, Hossein Shooli, Nastaran Hosseini, Anahita Fathi Kazerooni, Ariana Familiar, Sina Bagheri, Hannah Anderson, Stephen J. Bagley, Ali Nabavizadeh

**Affiliations:** 1 Center for Data-Driven Discovery in Biomedicine (D3b), Children’s Hospital of Philadelphia, Philadelphia, PA 19104, USA; nastarank@chop.edu (N.K.); fathikazea@chop.edu (A.F.K.); familiara@chop.edu (A.F.); 2 Department of Radiology, Bushehr University of Medical Sciences, Bushehr 75146-33196, Iran; 3 School of Medicine, Isfahan University of Medical Sciences, Isfahan 81746-73461, Iran; n.hosseini@res.mui.ac.ir; 4 AI2D Center for AI and Data Science for Integrated Diagnostics, University of Pennsylvania, Philadelphia, PA 19104, USA; 5 Department of Neurosurgery, Perelman School of Medicine, University of Pennsylvania, Philadelphia, PA 19104, USA; 6 Department of Radiology, Perelman School of Medicine, University of Pennsylvania, Philadelphia, PA 19104, USA; sina.bagheri@pennmedicine.upenn.edu (S.B.); hannah.anderson@pennmedicine.upenn.edu (H.A.); 7 Department of Medicine, Perelman School of Medicine, University of Pennsylvania, Philadelphia, PA 19104, USA; sbagley@pennmedicine.upenn.edu

**Keywords:** liquid biopsy, brain tumor, imaging, MRI, PET/MRI, cell-free DNA, circulating tumor DNA

## Abstract

**Simple Summary:**

Clinical management in neuro-oncology has shifted to an integrated method that combines molecular profiles with histopathological and imaging data. Liquid biopsy is a non-invasive method that captures the molecular diversity of the whole tumor by detecting specific tumor biomarkers that circulate in body fluids like the cerebrospinal fluid. However, the limited presence and short half-life of tumor-derived biomarkers, especially in central nervous system (CNS) tumors, have restricted the use of liquid biopsy in clinical settings. Here, we review the diverse clinical applications of liquid biopsy in CNS tumors and discuss the added value of imaging in enhancing the release and detection of circulating tumor biomarkers.

**Abstract:**

Clinical management in neuro-oncology has changed to an integrative approach that incorporates molecular profiles alongside histopathology and imaging findings. While the World Health Organization (WHO) guideline recommends the genotyping of informative alterations as a routine clinical practice for central nervous system (CNS) tumors, the acquisition of tumor tissue in the CNS is invasive and not always possible. Liquid biopsy is a non-invasive approach that provides the opportunity to capture the complex molecular heterogeneity of the whole tumor through the detection of circulating tumor biomarkers in body fluids, such as blood or cerebrospinal fluid (CSF). Despite all of the advantages, the low abundance of tumor-derived biomarkers, particularly in CNS tumors, as well as their short half-life has limited the application of liquid biopsy in clinical practice. Thus, it is crucial to identify the factors associated with the presence of these biomarkers and explore possible strategies that can increase the shedding of these tumoral components into biological fluids. In this review, we first describe the clinical applications of liquid biopsy in CNS tumors, including its roles in the early detection of recurrence and monitoring of treatment response. We then discuss the utilization of imaging in identifying the factors that affect the detection of circulating biomarkers as well as how image-guided interventions such as focused ultrasound can help enhance the presence of tumor biomarkers through blood–brain barrier (BBB) disruption.

## 1. Introduction

For the first time, in 1869, Ashworth hinted at the presence of circulatory cells in the blood of a patient with cancer that resembled the appearance of tumor cells [1]. Later studies reported the presence of short fragments of free DNA in the blood, nowadays termed cell-free DNA (cfDNA), which seemed to be found in higher levels in patients with cancer [2,3]. It is now widely known that many tumors shed their genetic and non-genetic material into biological fluids after undergoing necrosis or apoptosis. Examples of these tumoral contents include circulating tumor DNA (ctDNA), circulating tumor cells (CTCs), tumor-specific mRNA, microRNAs (miRNA), proteins, extracellular vesicles (EV), and the recently discovered tumor-educated platelets (TEP) [2]. “Liquid biopsy” is a term that refers to the detection and analysis of these tumor biomarkers through obtaining samples from different biological fluids. Although the most common source for liquid biopsy is plasma, based on the type of cancer, a variety of other body fluids such as saliva, urine, cerebrospinal fluid (CSF), pleural and peritoneal fluid, and even stool can be used to investigate tumor-specific biomarkers [3]. 

Regardless of the tumor type, liquid biopsy has the potential to be utilized as a minimally invasive method for tumor detection as well as for the real-time monitoring of disease and predicting progression. With the advent of genotype-directed therapies, liquid biopsy can also aid in unraveling the mechanisms of tumor resistance and advance the development of targeted therapies [4]. 

**Figure 1:**
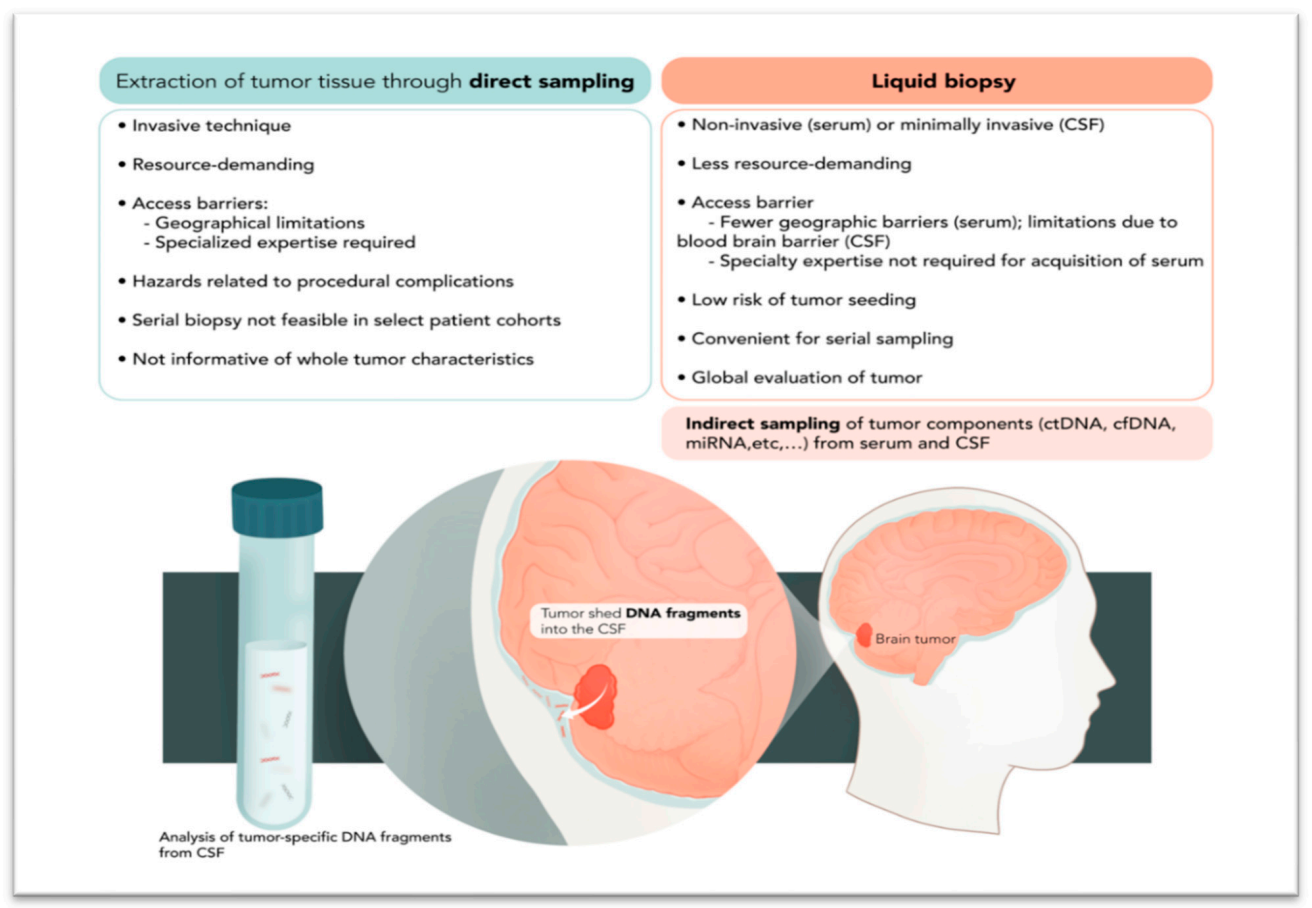
Advantages of indirect assessment (liquid biopsy) of tumor genome content compared with direct sampling (tissue biopsy).

Since the discovery of the significant prognostic impact of certain molecular alterations and the emerging role of therapeutically targeting these alterations in central nervous system (CNS) tumors, the molecular profiling of CNS tumors has become an integral component of routine neuro-oncologic care. Currently, the molecular profiling of CNS tumors is mainly achieved through invasive procedures such as tissue biopsy and surgical resection. In addition to being costly, these procedures can be associated with complications and are especially challenging for disseminated disease and tumors located in eloquent regions of the brain. Due to these reasons, liquid biopsy is an attractive option for the genetic profiling of CNS tumors [4]. Moreover, in comparison to conventional tissue sampling, liquid biopsy provides the opportunity to capture the complex heterogeneity of the whole tumor and provides information about global tumor characteristics, which is important given the prominent intra-tumoral heterogeneity of CNS tumors [5]. Not only has the genomic information obtained through liquid biopsy been found to closely match and correlate with respective glioma tumor tissue, in some instances, liquid biopsy has provided further information that was unidentifiable through tissue sampling [6]. Figure 1 summarizes the advantages of liquid biopsy compared with direct tumor sampling through tissue biopsy.

Despite all of the potential advantages, the low abundance of tumor-derived biomarkers in the blood of most patients with CNS tumors has limited the application of liquid biopsy in clinical practice [7]. It is, therefore, crucial to identify the factors that are associated with the presence of greater levels of these analytes and explore possible strategies that can increase the shedding of tumoral components into biological fluids. Here, we review the clinical applications of liquid biopsy in CNS tumors and discuss the role of imaging in augmenting the presence of circulating tumor biomarkers and better characterizing tumor evolution during the disease course.

## 2. Clinical Applications of Liquid Biopsy in Brain Tumors

### 2.1. Pre-Operative Setting: Cancer Screening, Early Detection, and Diagnostic Differentiation

In the context of CNS tumors, the CSF and plasma seem to be the most relevant sources for providing detailed insights into tumor mutational status, thus providing an opportunity for early tumor detection and non-invasive diagnostics. The molecular data detected in these fluids can potentially depict the existence of tumor prior to abnormalities being detected on MR imaging, allowing for earlier therapeutic intervention [8]. Increasing evidence suggests that tumor driver mutations and chromosomal alterations do not always occur randomly; in fact, in many instances, they demonstrate explicit patterns [9]. Liquid biopsy can aid in unveiling these patterns, opening up the potential for the early detection of disease in apparently healthy individuals. However, such a screening approach demands high sensitivity to detect minute concentrations of tumoral content released by precancerous or early-stage lesions, as well as excellent specificity for minimizing false positive results. Achieving high sensitivity in blood-based assays is particularly complicated in brain tumors because of the BBB and the relative absence of extra-CNS spread of tumor [10]. 

To date, many studies have aimed to evaluate the efficacy of liquid biopsy for discriminating patients with tumor from healthy controls. For instance, some studies have investigated the utility of methylated tumor-specific DNA for the early detection of brain tumors with different histologies (Table 1). Nonetheless, multiple challenges persist regarding the potential application of brain-tumor-derived circulating DNA as a diagnostic tool. For example, the utility of using cfDNA in the peripheral blood of patients with primary brain tumors for the simultaneous detection of various molecular alterations remains uncertain. A recent study in a cohort of patients with gliomas of various grades evaluated the efficacy of serum DNA for the concurrent detection of the loss of chromosomal heterozygosity and O(6)-methylguanine-DNA methyltransferase (MGMT) methylation. Serum-derived ctDNA demonstrated an overall moderate sensitivity for the detection of both allelic deletions and methylation profile; however, the specificity was very high, approaching almost 100% [11]. Another study in patients with meningioma also showed that the level of DNA methylation detected through the analysis of plasma cfDNA is significantly correlated with the grade of the tumor [12]. Utilizing CSF-derived ctDNA for epigenetic profiling, including studying DNA methylation and hydroxymethylation, has also been studied in pediatric tumors such as medulloblastoma [13]. 

Beyond cfDNA, RNAs and EVs have also been studied in brain tumors as potential liquid biopsy analytes. For example, decreased levels of glioma-specific miRNAs, including miR-15b, miR-23a, miR-133a, miR-150, miR-197, miR-497, and miR-548b, have been observed in the serum of patients with glioma compared with healthy controls, proposing them as possible biomarkers for diagnosis. The expression of miR-21 was also found to be higher in patients with glioblastoma compared to the normal population [14]. Likewise, EVs detected in the CSF and/or plasma can also provide valuable diagnostic information for patients with glioblastoma. Not only does the level of EVs in the plasma convey information about the presence of tumor, but it also reveals useful prognostic information regarding the status of molecular alterations such as EGFR amplification, PTEN deletion, and IDH1/2 and TP53 mutations [15]. The most notable advantages of EVs are that they can cross anatomical hurdles such as the BBB and provide protection for tumoral contents from being degraded by circulatory enzymes [16]. A very novel way to capture tumor-derived EVs is through TEPs; extracted TEPS were able to demonstrate EGFRvIII mutation in 80% of patients with glioblastoma [17]. 

Evidence suggests that in patients with primary CNS malignancy, the tumor components are more abundant in the CSF than in the plasma, suggesting that the CNS can serve as a higher-fidelity source for liquid biopsy in CNS tumors. Studies have shown the value of cfDNA extracted from the CSF in identifying somatic mutations such as MGMT, p16INK4a, TIMP-3, and THBS1 that can only be detected in patients with glioblastoma and not healthy individuals [5]. Similarly, EVs and miRNAs extracted from the CSF show high sensitivity and specificity for distinguishing glioblastoma from normal non-cancer controls [18]. Larger, prospective studies are required to identify and eventually validate CSF biomarkers for routine clinical use. 

### 2.2. Identification of Post-Operative Tumor Residual and Progression Surveillance

Given the very short half-life of ctDNA (less than one hour), the detection of ctDNA in the post-operative plasma following curative surgery for patients with cancer can provide valuable evidence regarding the presence of minimal residual disease. Thus, the persistent presence of tumoral components detected by liquid biopsy weeks after surgery or chemoradiation can suggest a higher likelihood of eventual tumor recurrence [19]. The information provided through liquid biopsy can aid in the early stratification of patients based on their risk of recurrence and provide an opportunity for early intervention and the potential escalation of therapy.

In patients with glioblastoma, distinguishing between pseudoprogression and true tumor progression is a notable challenge. Misinterpreting these conditions can have serious consequences, including the early termination of an effective treatment or overestimating the effectiveness of subsequent salvage therapies. While advanced magnetic resonance imaging (MRI) methods like dynamic susceptibility contrast (DSC), dynamic contrast-enhanced (DCE), and diffusion-weighted imaging (DWI) have enhanced the capacity to distinguish pseudoprogression from true progression, substantial inconsistencies in acquisition and analysis methods across different institutions has hindered their use in both clinical practice and research. Moreover, these techniques exhibit suboptimal accuracy and are often susceptible to imaging artifacts in the post-treatment setting [20]. In the context of progression surveillance, studies have been performed that exhibit the value of tumor biomarkers detected through liquid biopsy in predicting tumor grade and overall prognosis for CNS tumors. A recent study reported that the epigenome-wide methylation of cfDNA in the serum of patients with glioma acts as a highly specific and sensitive marker for the detection of glioma. The authors developed a score metric called the “glioma-epigenetic liquid biopsy score”, or GeLB, that was able to distinguish patients with or without glioma. In addition, the quantitative assessment of cfDNA correlated with dynamic clinicopathological changes during surveillance, including during progression and response to therapy [21]. Another prospective study also described the prognostic utility of plasma cfDNA as a surrogate of tumor burden and progression in patients with glioblastoma; a longitudinal assessment of 12 post-operative cases showed that there was no association between plasma cfDNA concentration and tumor burden at the time of radiation (RT) simulation. Still, a significant correlation was observed between plasma cfDNA concentration and tumor volume at 1-month post-RT [22]. Another study indicated that because of the short half-life of circulating cfDNA, the continuous presence of tumor-specific DNA in the circulation of patients with glioma is probably indicative of residual tumor rather than the post-treatment shedding of DNA fragments into the bloodstream; this finding was supported by the significant correlation between positive serum-derived DNA and the presence of a measurable tumor on recently performed MRI studies [11]. 

In addition to DNA-based assays, various studies have also been performed to evaluate the prognostic role of glioma-specific miRNAs derived through liquid biopsy. For instance, studies suggest that the decreased serum level of multiple miRNAs such as miR-125b, miR-497, miR-205, miR-128, and miR-342 is indicative of a higher histopathological grade in patients with glioma [23]. Conversely, the serum level of miR-21, miR-221, miR-222, miR-210, and miR-182 is higher among patients with high-grade glioma and correlates with poor survival [24,25]. Plasma haptoglobin α2 is a novel tumor biomarker that has shown capability in distinguishing glioblastoma from low-grade glioma [26]. Likewise, elevated serum YKL-40, AHSG, α-tocopherol, and γ-tocopherol levels appear to be increased in glioblastoma patients and are associated with unfavorable prognosis and lower overall survival [27]. Also, the plasma levels of circulating metabolites such as arginine, methionine, and kynurenate could predict prognosis in patients with glioblastoma [28]. In summary, liquid biopsy has the potential to provide early, non-invasive evidence of response or progression and contribute to better clinical management. 

### 2.3. Selection of Precision Therapies and Understanding Mechanisms of Resistance

Despite significant variations in ctDNA levels across patients with glioma, these biomarkers correlate well with temporal changes of tumor burden in an individual patient and can be used as biomarkers for the dynamic monitoring of treatment response in this patient population [6]. Furthermore, since the analysis of tumoral components has proved effective in identifying emergent mutations, liquid biopsy could possibly be used to identify mechanisms of therapeutic resistance and, subsequently, guide treatment selection. In fact, liquid biopsy has already yielded promising results in investigating the mechanisms of resistance in several cancers, including non-small cell lung cancer, colorectal and metastatic breast cancer [29,30,31]. As the current treatment strategies for patients with CNS tumors become increasingly dependent on the presence or absence of specific molecular markers, including when tumors relapse after standard first-line treatment, the role of liquid biopsy in precision neuro-oncology is likely to expand in the near future. A key advantage of liquid biopsy is its capability to uncover the molecular heterogeneity associated with therapeutic resistance in different tumor subclones.

A significant clinical challenge persists in the growth of tumors within the CNS even when systemic disease control is achieved. In this regard, CSF genomic profiling through cfDNA has been used for understanding drug resistance mechanisms in patients with progressive metastatic CNS involvement whose primary tumor responded to targeted cancer therapy. For instance, epidermal growth factor receptor (EGFR) T790M, KRAS G12A, and BRAF V600E mutations were found in the CSF-derived cfDNA of patients with metastatic lung cancer and melanoma who had initially responded to kinase inhibitors [32] (also see Table 1 and Table 2).

Another novel area of interest, specifically regarding immunotherapy, is monitoring the interaction between EVs and the immune system as EVs can exchange signals between the brain cells and the surrounding stroma and alter the tumor immune microenvironment [33,34]. As Programmed death-ligand 1 (PD-L1) expression has been found on the surface of glioblastoma-derived EVs, the presence of PD-L1 can indicate resistance to immune checkpoint inhibitors such as anti-PD-1 [35]. Besides this, EVs harboring specific mutations, such as MGMT or transglutaminase 2 (TGM2), have been shown to confer resistance to temozolomide (TMZ). Elevated levels of EVs expressing surface molecules such as CD44 and CD133 were also shown to be associated with TMZ failure in patients with glioblastoma [36]. Similarly, cultured CTCs derived from the serum of patients with glioblastoma have been shown to express glioma stem cell markers such as SOX2, OCT4, and NANOG that drive resistance to radiotherapy or TMZ [37]. These findings have also led to efforts to use liquid biopsy in clinical trials; for example, liquid biopsy has been used to monitor epidermal growth factor receptor variant III (EGFRvIII) status and assess the treatment response in patients with glioblastoma vaccinated with rindopepimut [38]. Table 1 and Table 2 show a list of studies that have explored different applications of liquid biopsy across a variety of adult and pediatric CNS tumors, respectively. 

**Table 1:** Select studies showing the role of liquid biopsy in different adult CNS tumors.

Histopathology	Biopsy Source	Tumoral Content	Molecular Alterations Studied	Isolation Technique	Application/Findings
**GBM**					
[39]	Serum	cfDNA	MGMT, p16, DAPK, RASSF1A methylation	MS-PCR	Correlation with time to progression and response to 1,3-bis(2-chloroethyl)-1-nitrosourea (BCNU) and temozolomide
[40]	Plasma	ctDNA	P16, MGMT, p73, and RARβ methylation	MS-PCR	Identification of tumor-specific promoter methylation
[41]	Urine	Panel of 23 miRNAs	-	Nanowire	Screening method for early detection of tumor
[42]	Neurosurgical aspirate fluid	EVs, miR-486	-	NGS	Distinguishing GBM from Lower-Grade Astrocytoma
**LGG**					
[43]	Serum	miR-21, miR-20e, miR-223	-	ddPCR	Post-operative monitoring
[6]	CSF	ctDNA	DH1, 1P19Q, CIC, ATRX, TP53 mutation	NGS	Monitor evolution of the glioma genome through disease course Correlation with disease burden
**Meningioma**					
[44]	Serum	ctDNA	MGMT, RASSF1A, p15INK4B, and p14ARF methylation	MS-PCR	RASSF1A hypermethylation differentiates between metastatic and primary CNS cancers two groups
[45]	Plasma, CSF	cfDNA	NF2, AKT1 mutation	dd-PCR	Higher cfDNA concentrations in CSF than in plasma; CSF may be used for disease detection despite low plasma cfDNA concentrations
[35]	Plasma	EVs	22q and 1p deletion, NF2 and TRAF7 mutation	Nanoparticle tracking analysis	Tumor detection and classification, pre-operative tumor assessment and residual tumor monitoring, correlation with tumor size, grade and peritumoral edema
[46]	Serum	miR-15a, miR16_1, miR−15b, miR-497, miR-195	-	qPCR	Differentiating low-grade from high-grade meningioma
[47]	Serum	miRNA 200a, miRNAs 34a, miRNA 409	Aberrations of parts of chromosomes 1, 14, 18, and 22	qPCR	Predicting recurrent meningiomas

GBM: glioblastoma multiforme; MS-PCR: Methylation-specific PCR; dd-PCR: digital-droplet PCR; qPCR: quantitative-PCR; NGS: next-generation sequencing; miRNA: microRNA.

**Table 2:** Selected studies showing the role of liquid biopsy in different pediatric CNS tumors.

Histopathology	Biopsy Source	Tumoral Content	Molecular Alteration Studied	Isolation Technique	Application/Findings
**DMG/DIPG/HGG**					
[48]	Plasma, CSF, cystic fluid	ctDNA	H3K27M, IDH1, BRAF, MYCN	dd-PCR	Increased cfDNA concentrations was associated with shorter time to progression in DIPG and, conversely, better survival in HGG patients, tumor-specific DNA alterations more readily identified in CSF than plasma
[49]	CSF, Plasma, cystic fluid	ctDNA	H3K27M	dd-PCR	Assessing response to radiotherapy and recurrence
[50]	CSF	ctDNA	H3K27, H3.3G34	PCR	Detecting mutations
[51]	CSF, blood	ctDNA, cfDNA	H3K27	dd-PCR	Predicting recurrence prior to imaging, predicting response to therapy, differentiating progression and pseudoprogression
**Medulloblastoma**					
[13]	CSF	ctDNA	CTNNB1, SUFU, KMT2D, CREBBP, KBTBD4, PT53, DDX3X, PTCH1 KDM6A	qPCR	Detection of different methylation patterns, metastasis status, correlation with tumor burden and location, prediction of disease progression, evolution of the genome in response to therapy
[52]	CSF, blood	ctDNA	KMT2D, KMT2C, SMARCA4, BCOR, TP53, PTCH1, EP300, NF1, SETD2, MED12, SPEN	qPCR	ctDNA correlated with disease progression and metastasis; tumor-specific alterations detected more frequently in CSF than tumor tissue
[53]	CSF	ctDNA	TP53, PTEN, PTCH1, BCOR mutation, 17p deletion	qPCR	Assessing minimal residual disease and tumor evolution, identifying intra- and intertumoral heterogeneity
[54]	CSF	cfDNA	CpG methylation	qPCR	Detecting tumor and its subtype, monitoring treatment response and recurrence

DMG: diffuse midline glioma; DIPG: diffuse intrinsic pontine glioma; HGG: high-grade glioma.

## 3. Role of Imaging in Liquid Biopsy of Brain Tumors

As previously discussed, the plasma concentration of cfDNA is lower in patients with CNS tumors than other solid tumors, leading to the decreased probability of ctDNA detection using blood-based assays. Although the advent of new technologies such as droplet-based digital PCR (dd-PCR) or next-generation sequencing (NGS) has improved the sensitivity for detecting ctDNA mutations, these assays do not yet have high enough sensitivity for routine implementation in the blood of patients with CNS tumors. It is thought that the release of CNS tumor-derived biomarkers into the peripheral circulation is limited by the BBB and other factors [25]. Thus, understanding the factors that influence the detection of circulating tumor biomarkers may lead to a more efficient use of liquid biopsy in the clinic. In this section, we discuss the role of imaging in improving tumor biomarker detection and how it can contribute to the broader application of liquid biopsy in patients with CNS tumors.

### 3.1. Identifying Factors That Affect Plasma cfDNA and ctDNA Detection

One recent study attempted to identify the association between plasma cfDNA concentration and the radiographic tumor burden of patients with glioblastoma at different time points before and after receiving adjuvant chemoradiation treatment [22]. The investigators of this study assessed the correlation between total plasma cfDNA concentration and total radiographic tumor burden, defined as the sum of the volumes of T1 post-contrast enhancing tumor and T2/fluid-attenuated inversion recovery (FLAIR) signal abnormality on MRI, as well as each of these volumes (contrast-enhancing tumor or T2/FLAIR signal abnormality) in isolation. The results indicated a significant correlation between plasma cfDNA concentration at 1-month post-radiation and both total tumor volume and contrast-enhancing tumor volume. However, no meaningful association was observed between the plasma cfDNA concentration and total radiographic tumor burden, contrast-enhancing tumor volume, or T2/FLAIR signal abnormality at the pre-operative and radiation stimulation timepoints [22]. Thus, it is likely that factors beyond tumor volume contribute to the release of cfDNA into the circulation. 

Further investigations have shed light on the possible role of features such as BBB integrity and peritumoral immune cell density in the detection rate of circulating cfDNA in patients with GBM. A recent study of patients with treatment-naive GBM utilized advanced MR imaging sequences, including diffusion tensor imaging, dynamic contrast-enhanced (DCE) perfusion, and dynamic susceptibility contrast perfusion, to assess the relationship between the various imaging measures of BBB permeability, tumor vasculature, and tumor cellularity and plasma cfDNA and ctDNA concentrations [55]. This study demonstrated a positive correlation, with the volume of tumor displaying elevated *K*_trans_ and elevated *K*_ep_ metrics, which are considered surrogates of BBB permeability, and plasma cfDNA concentration. Additionally, the histopathologic analysis of the same subset of patients demonstrated that a higher perivascular CD68+ macrophage density is associated with a significantly lower volume transfer constant (*K*_trans_), as quantified by DCE MRI, which is hypothesized to be due to the anti-inflammatory nature of tumor-associated macrophages [55]. These findings strengthen the rationale for the potential effect of lower BBB permeability on the decreased detection rate of somatic mutations in the plasma specimens of patients with brain tumors. 

Another interesting aspect of correlating imaging findings with liquid biopsy is in the context of identifying post-treatment disease progression, as brain MRI has a limited ability in differentiating true tumor progression from pseudoprogression. The longitudinal follow-up of a cohort of patients with GBM showed that an increase in total plasma cfDNA levels following first-line chemoradiotherapy compared to the pre-treatment baseline was associated with markedly worse PFS and OS, even if the first post-radiotherapy MRI scan did not show tumor progression [22]. Another study longitudinally assessed the serum level of hypoxia-mediated microRNAs that are upregulated in glioma (miR-21 and miR-10b) before and after treatment with bevacizumab. They observed that in patients with enhancing tumor, the miR-10b and miR-21 levels have a significant negative correlation with changes in the diameter of the enhancing tumor. This negative correlation was also displayed between miR-10b plasma levels and changes in FLAIR measurements in patients with non-enhancing tumor [56]. 

Other studies have evaluated imaging in relation to CSF liquid biopsy. One study of 85 previously treated patients with glioma aimed to identify the radiological correlates of ctDNA shedding into the CSF [6]. In this study, which included patients with both low-grade and high-grade tumors, 42/85 patients (49%) had at least one tumor-derived genetic alteration in the CSF and were considered to be patients with positive CSF ctDNA. Findings such as the presence of enhancing core, tumor burden, and radiographic progression (based on RANO criteria) were assessed using standard brain MRI sequences (T1-weighted, T2-weighted, FLAIR, and contrast T1-weighted images) and were compared between ctDNA-positive and ctDNA-negative patients. Imaging evidence of disease extension into the subependymal, pial, and subarachnoid space, which served as a surrogate marker of tumor spread into the CSF, was also investigated. Based on the results of this study, tumor progression, tumor burden, and the ventricular or subarachnoid spread of the tumor were significantly associated with the presence of ctDNA in the CSF. Interestingly, the presence of an enhancing component did not demonstrate a remarkable association with an increased release of ctDNA. In this study, Miller and colleagues also tested the presence of mutations in the plasma of 19 patients with positive CSF ctDNA. Mutations were detected in the plasma of only 3/19 (16%) patients. Noticeably, all three patients with positive plasma ctDNA demonstrated imaging evidence of disseminated disease within the CNS [6]. This finding suggests that in glioma patients, the CSF acts as a more sensitive reservoir for the detection of tumor biomarkers compared with plasma.

In an exploratory study, Wang and colleagues used MR imaging to assess the relationship between the anatomical location of brain tumors and the detectability of tumor-derived biomarkers in the CSF. Although there was no marked difference between supratentorial, infratentorial, and spinal tumors, lesions adjacent to a CSF reservoir in the brain or spinal cord were more likely to have detectable levels of tumor DNA in the CSF [57]. Another study demonstrated that the number of lesions on enhanced MRI affects miR-10b expression levels, with higher levels being detected in patients with multiple lesions compared with single lesions [58].

### 3.2. Enhancing Presence of Tumor Biomarkers through Blood–Brain Barrier (BBB) Disruption

**Figure 2:**
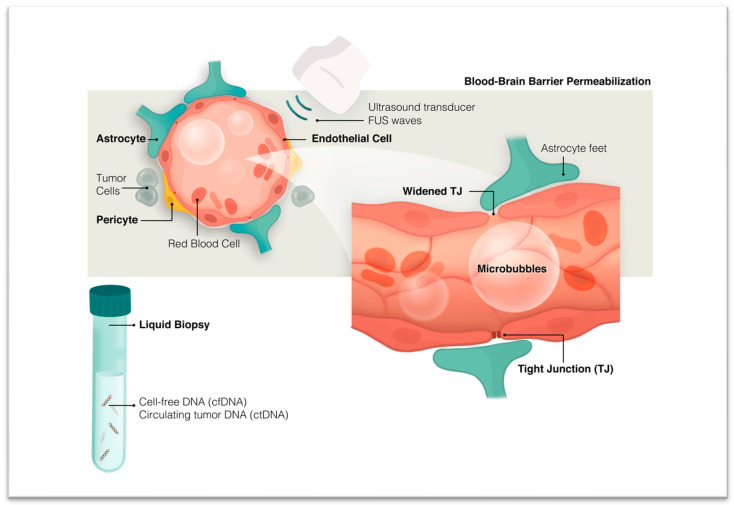
Targeted disruption of the blood–brain barrier through focused ultrasound.

As discussed in the previous section, the BBB seems to act as a functional and structural barrier in the release of brain tumor biomarkers into the circulation. Due to the numerous benefits of liquid biopsy, exploring non-invasive approaches that can induce transient BBB opening and enhance the release of brain-tumor-derived biomarkers into the circulation is of utmost importance. Since the initial discovery of its biological effect, focused ultrasound (FUS) has been used as a tool for ablating tumor tissue and enhancing drug delivery through the targeted disruption of the BBB (Figure 2). The many advantages of FUS, such as non-invasiveness, high temporal resolution, and reversibility, make it a promising tool for increased access to the brain, both for the delivery of therapeutics and tumor-derived biomarker detection. High-intensity focused ultrasound (HIFU) can significantly increase tissue temperature, thus providing the therapeutic opportunity for the selective thermocoagulation of brain tumors [59,60]. 

On the other hand, in combination with microbubbles (MB), a lower intensity of ultrasound energy can be used for the specific purpose of increasing the permeability of the BBB [61]. In this method, optimal ultrasound acoustic pressures cause the contraction and expansion of intravenously introduced MBs, which, in turn, exert mechanical forces and produce sheer stress on the brain microvascular membrane. The ultrasonic force also promotes MB and BBB interaction by directing oscillating MBs to the endothelial cell membrane. This process, commonly referred to as cavitation, allows for reversible BBB opening through the disruption of tight junctions and enhanced BBB permeability [62]. In comparison with HIFU, low-intensity ultrasound has a better safety profile, preventing complications such as hemorrhage or normal tissue damage [63]. However, other parameters such as pulse-repetition frequency, microbubble size, and pulse duration also need to be adjusted to control the extent of ultrasound-mediated BBB disruption [63]. Several studies have evaluated the efficacy of low-intensity FUS for increasing the release of plasma cfDNA and have explored the optimal time point for collecting markers following sonication. However, recent pre-clinical and clinical studies have shown that FUS is only able to increase BBB permeability for up to 24 h post-sonication [64]. Additionally, the release of biomarkers was found to be time-dependent, with significant increases in cfDNA concentrations starting 15 min after sonication, peaking at 60 min [65]. More recent efforts have focused on MRI-guided focused ultrasound (MRgFUS) for delivering targeted acoustic energy and the selective disruption of the BBB, hence increasing the release and detection of brain tumor biomarkers [66,67,68]. In a recent study on a murine GBM model, by applying low-intensity FUS, the sensitivity for detecting EGFRvIII and TERT C228T mutations increased significantly 10 min after sonication; specifically, the detection sensitivity of the EGFRvIII mutation reached 100% in the porcine model of GBM [69]. MRgFUS did not significantly increase the risk of microhemorrhages, and only minimal off-target damage was observed [69]. A first-in-human proof-of-principal trial of low-frequency MRgFUS was conducted on nine patients with GBM receiving adjuvant TMZ. The concentration of plasma cfDNA, S100b, and neuron-derived extracellular vesicles (as measured by NCAM and L1CAM expression) demonstrated a 2.6-, 1.4-, and 3.2-fold increase, respectively, after approximately 30 min following the last sonication; this increase displayed a positive correlation with treated volume and a weak negative correlation with time following sonication. As with pre-clinical studies, no serious adverse events were reported in any of the patients [68]. Overall, the FUS-enhanced disruption of the BBB represents a new era in the liquid biopsy of brain tumors, with the potential for great progress in both diagnostic and therapeutic aspects.

### 3.3. Role of Advanced Imaging Techniques in the Clinical Setting

Given the current limitations of liquid biopsy, its application in the clinical setting is still challenging. Therefore, there is a need for complementary techniques along with liquid biopsy to address shortcomings and improve accuracy. There is a potential to combine liquid biopsy with novel imaging techniques such as molecular imaging techniques with positron emission tomography (PET) or radiomic techniques to address the current limitations.

#### 3.3.1. Advanced MRI Techniques in Combination with Liquid Biopsy 

**Figure 3:**
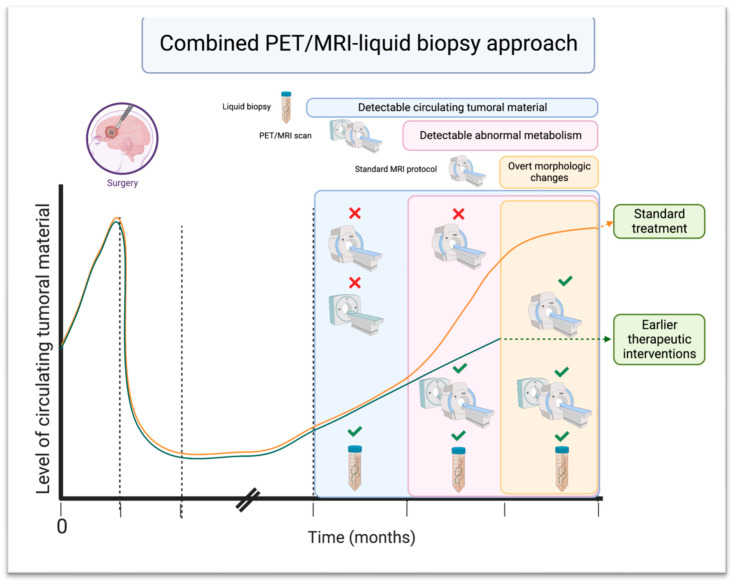
Proposed combined PET/MRI–liquid biopsy approach. This approach offers a comprehensive assessment of genetic profile, metabolic alterations, and morphological changes in brain tumors in the post-operative setting and has the potential to monitor dynamic changes in brain tumors.

Currently, standard practice for the surveillance of post-operative tumor recurrence relies on detecting morphological changes via MRI; however, this approach has low accuracy for distinguishing between tumor progression and treatment-related changes [70]. Recently, quantitative MRI methods such as dynamic contrast-enhanced MRI, dynamic susceptibility contrast MRI, MR spectroscopy, and diffusion MRI have gained attention to improve the imaging of brain tumors. These techniques have the potential to provide valuable information for tumor characterization and help yield information about tumor type, grade, response to therapy, and pseudoprogression [71]. However, these MRI techniques also have several limitations; liquid biopsy might have the ability to address these limitations when used as a complementary tool. For example, the longitudinal assessment of perfusion parameters is difficult, as the co-registration of subsequent follow-up scans might introduce errors, but the simultaneous application of liquid biopsy can help confirm the findings [72]. Another modality, PET/MRI, is beneficial in evaluating brain tumors since it can visualize biochemical and physiological processes alongside anatomical details. Real-time MRI measurements of microvascular proliferation and permeability simultaneously with PET tracer uptake helps quantify tumor proliferation, tumor vascular properties, and antitumor effects. PET/MRI can detect metabolic alterations that occur before morphological changes and is capable of utilizing various radiotracers to visualize different brain biological processes, tailored to individual clinical scenarios [73]. To date, the different PET radiotracers that have been used to evaluate brain tumors include fluorine-18 fluorodeoxyglucose (^18^F-FDG), carbon-11 methionine (^11^C-methionine), fluorine-18 fluoroethyltyrosine (^18^F-FET), fluorine-18 fluorodihydroxyphenylalanine (^18^F-FDOPA), fluorine-18 fluorothymidine (^18^F-FLT), and radiolabeled choline (^11^C-choline or ^18^F-choline) [74]. These tracers can be used for unique purposes such as glioma grading, detecting recurrent brain tumors, brain metastases, and even primary CNS lymphoma [74,75]. For instance, the combination of amino acid PET and advanced MRI has enabled the identification of molecular alterations such as IDH mutant status [76]. Integrating PET/MR with liquid biopsy might have the potential to further enhance the understanding of tumor biology, evolution, and therapeutic response on an individual basis. PET/MRI could potentially decrease the rate of false positive results in conventional MRI scans by detecting actively metabolic tumor lesions. This is especially advantageous when dealing with cases where the tumor burden is minimal. Also, this technique can offer additional value in distinguishing benign and malignant brain lesions [73]. Liquid biopsy can identify genetic alterations that precede metabolic changes, enabling even earlier detection [77]. When used as complementary tools, PET/MRI and liquid biopsy might be able to assess genetics, metabolism, and morphology, particularly when standard imaging studies are inconclusive. This combined approach may enhance the understanding of the disease course and guide clinical decisions for the individualized management of patients. Figure 3 outlines the proposed combined PET/MRI–liquid biopsy approach for brain tumors. The clinical utility of integrating cfDNA and metabolic tumor burden findings has been investigated in the context of multiple other cancers. In one study, the combined utilization of cfDNA and PET/CT demonstrated superior capability in discriminating early NSCLC from tuberculosis compared to when each technique was used individually. Moreover, among early NSCLC patients, a positive correlation between cfDNA and SUV-max became apparent, which was not observed in healthy controls or tuberculosis patients [78]. Similarly, other studies have shown the relationship between cfDNA concentration and FDG PET/CT-derived parameters of patients with NSCLC [79]. Another study demonstrated high sensitivity and specificity for the detection of progression and treatment response in patients with follicular lymphoma using combined PET/CT and liquid biopsy, recommending consideration of this combinational approach in future clinical trials [80].

#### 3.3.2. Integrating Radiomics with Liquid Biopsy

It is being increasingly recognized that optimal cancer management requires a synergistic multi-omics approach. This includes collecting and integrating data from genomic, immunomic, proteomic, and radiomic databases. Radiomics and liquid biopsy are both minimally invasive tools that provide valuable quantitative information for detecting tumors and monitoring evolution, making them an attractive combination for both diagnosis and treatment decision making. In addition, distinct imaging features and gene expression data can be linked to develop a specific radiogenomic signature for predicting prognosis. This combination is particularly needed in tumors such as gliomas, which demonstrate vast spatial and temporal intra- and inter-tumoral heterogeneity. Several studies have been undertaken in solid tumors other than glioma to identify radiogenomic expression patterns that have prognostic and therapeutic significance through integrating radiomics and liquid biopsy. For example, in a recent prospective study, difference entropy and normalized inverse difference, which are indicative of a more homogeneous attenuation pattern on CT images, were associated with detectable ctDNA TP53 mutations and stagnant changes in cfDNA concentration in the early treatment period of patients with locally advanced lung cancer. In another study on patients with metastatic melanoma, a significant association was observed between several radiomic features and ctDNA mutant allele fraction (*maf)* levels [81]. These promising results pave the way for conducting similar studies in patients with brain tumors with the aim of understanding tumor evolution and advancing patient-tailored treatment strategies.

## 4. Challenges and Future Directions

Combining liquid biopsy and imaging as two non-invasive diagnostic techniques is attractive, but not without challenges. One of the challenges is that the commonly used technique to measure the disruption of the BBB is based on the extravasation of low-molecular-weight gadolinium MRI contrast agents, which may not necessarily correlate with the extravasation of liquid biopsy biomarkers [82]. Performing advanced imaging techniques, such as imaging of the glymphatic system, might help detect the shedding and release of tumor biomarkers into the circulatory system [83]. In addition, given that necrotic cells may be more likely to release tumor-specific DNA into the circulation compared with apoptotic cells [84], another future synergistic direction could be to focus on identifying the imaging biomarkers of necrosis. Furthermore, as described above, integrating other imaging techniques, such as PET/MRI or radiomics, may serve as a complementary tool for optimizing circulating tumor biomarkers.

## 5. Conclusions and Summary

In the era of precision oncology, understanding the genetic complexity of brain tumors and individualized molecular profiling is key to successful patient management. In this review, we discussed the advantages of liquid biopsy for the detection, diagnosis, genomic profiling, and monitoring of brain tumors. We also described the added benefit of imaging in identifying factors that are related to tumor biomarker release, as well as its utilization in amplifying the shedding of these biomarkers. More novel combinational approaches include using PET imaging and radiomics as complementary non-invasive tools that may provide a deeper characterization of brain tumors. Overall, imaging can provide added value to liquid biopsy for detecting tumor evolution, and together, these minimally invasive and cost-effective techniques can provide robust methods for optimizing personalized care in future clinical practice.
